# Ferromagnetism in two-dimensional metal dibromides induced by hole-doping

**DOI:** 10.1038/s41598-023-37777-8

**Published:** 2023-07-17

**Authors:** Ruishen Meng, Michel Houssa

**Affiliations:** 1 Department of Physics and Astronomy, KU Leuven, Leuven, 3001 Belgium; 2 IMEC, Leuven, 3001 Belgium

**Keywords:** Two-dimensional materials, Magnetic properties and materials, Electronic structure

## Abstract

Using spin-polarized first-principles calculations based on density functional theory, we study the stability, electronic properties and magnetic behavior induced by hole-doping of two-dimensional (2D) PbBr_2_ and HgBr_2_. Although inherently nonmagnetic, these materials can exhibit stable ferromagnetic order when hole-doped at densities above a few 10^13^ cm^-2^. We also examined the impact of intrinsic and extrinsic defects on inducing hole-doping and subsequent ferromagnetism. Our findings suggest that *p*-type doping can be achieved by Pb and Hg vacancies and Br antisites, but the latter behaves as deep acceptors. Among the possible dopants we considered, Li substituting Pb or Hg, and S replacing Br in 2D HgBr_2_, can produce shallow acceptor states near the valence band edges and potentially result in a stable ferromagnetic order in these 2D dibromides.

## Introduction

Over the past few decades, two-dimensional (2D) materials^1–3^, including graphene, other single-element 2D Xenes (e.g., X = Si, Ge, Sn, B, P, As, Sb), transition-metal dichalcogenides, as well as II–VI and III–V 2D compounds, have attracted significant research interest due to their exotic physical properties and potential use in various applications^4–6^, such as solar cells, electrocatalysts, photocatalysts, and nanoelectronic devices. Recently, 2D magnetic materials have also gained increased attention^7,8^. However, intrinsic 2D magnetic materials are scarce, and further effort is needed to continuously discover new candidates or induce magnetism into nonmagnetic materials.

**Figure 1:**
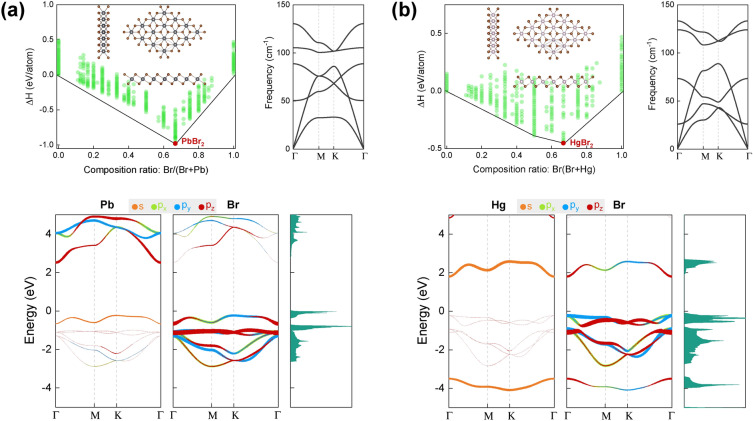
Convex hulls of different stoichiometries searched by USPEX, and the corresponding atomic structures of the most energetically favorable candidate (insert), phonon dispersion spectrum, orbital projected band structures and density of states of (**a**) 2D PbBr_2_ and (**b**) 2D HgBr_2_.

**Figure 2:**
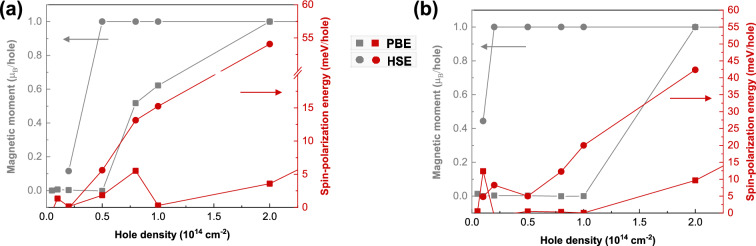
Magnetic moment and spin-polarization energy as a function of hole-doping density, calculated by PBE and HSE06 functional for 2D (**a**) PbBr_2_, and (**b**) HgBr_2_.

It has been demonstrated that point defects can induce a nonmagnetic to ferromagnetic phase transition in some materials. For instance, in the dilute magnetic semiconductor (DMS) Mn-doped GaAs, a ferromagnetic order can be observed up to approximately 180 K^9,10^. The Mn impurities provide both localized spins and delocalized holes simultaneously, and a long-range spin-spin interaction can be achieved through the mediation of the delocalized holes. Other DMSs, such as *p*-type GaN and ZnO, obtained through magnetic or even nonmagnetic doping, exhibit ferromagnetism above room temperature^11–13^. Furthermore, some intrinsic nonmagnetic semiconductors, such as TiO_2_, HfO_2_, and In_2_O_3_, show a Curie temperature above room temperature without any extrinsic doping^14^, with metal vacancy defects believed to be the origin of the ferromagnetism^15^. Although some of these results are controversial^16,17^, they highlight the benefits of hole mediation in promoting ferromagnetic order in these materials.

**Figure 3:**
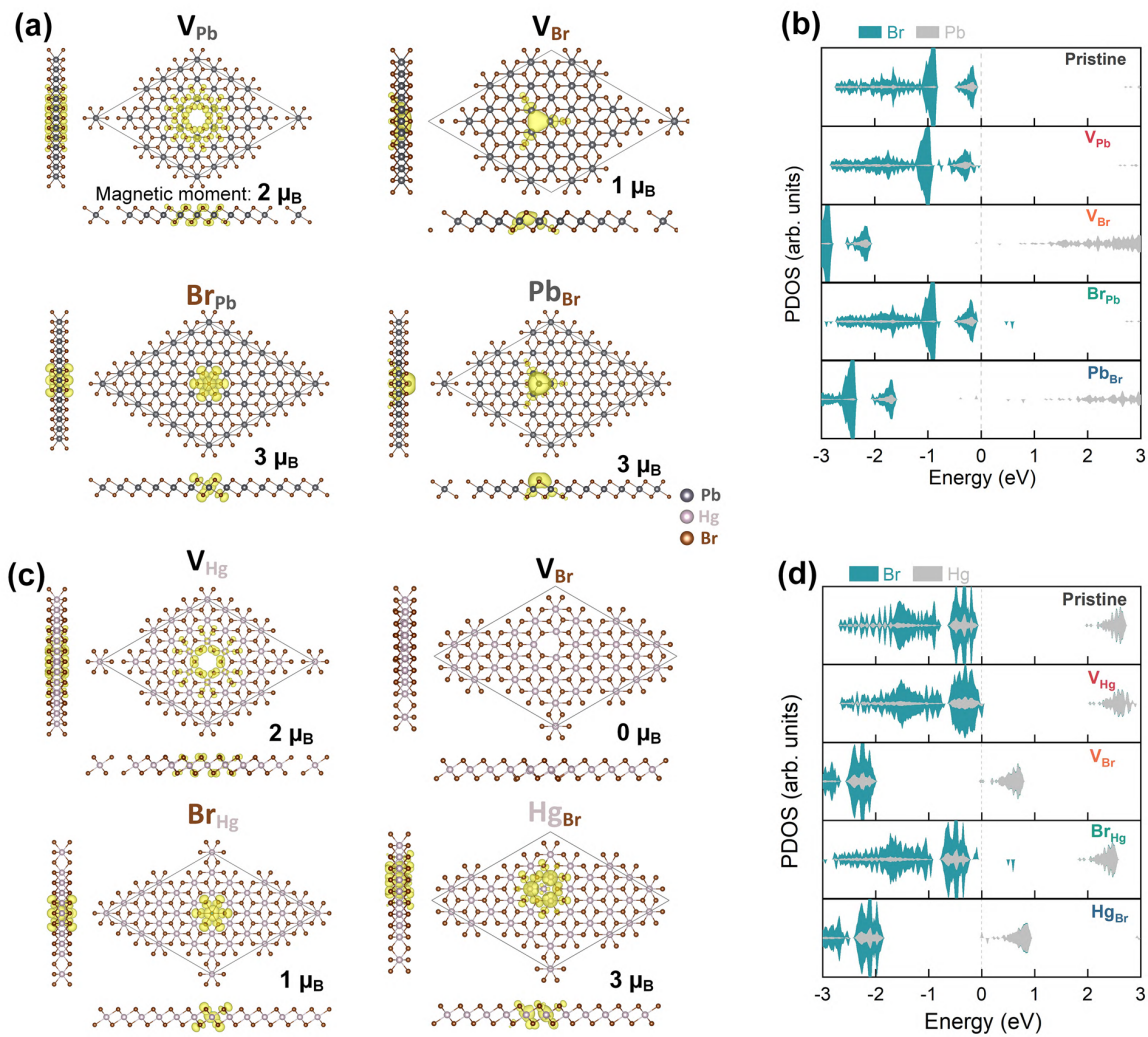
Relaxed atomic structures and Projected density of states of (**a**) and (**b**) 2D PbBr_2_, and (**c**) and (**d**) 2D HgBr_2_, respectively, with different intrinsic defects. The spin densities of each intrinsic defect are shown in yellow (spin-up) and cyan (spin-down), and the corresponding magnetic moment is also provided.

**Figure 4:**
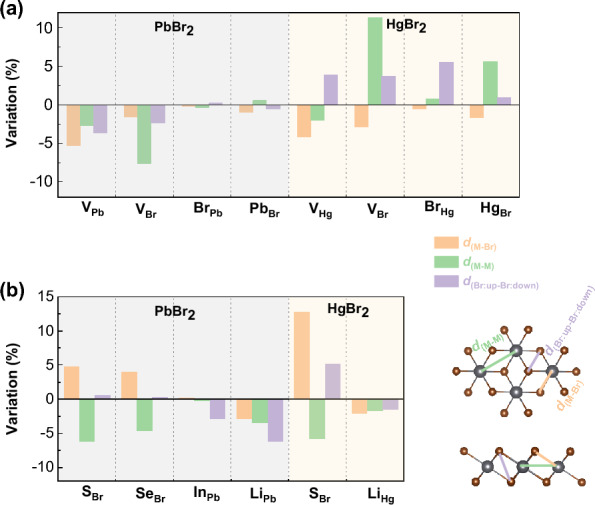
The maximum variation rate of the distances between the metal and the Br atoms (*d*_M-Br_), metal and metal atoms (*d*_M-M_), as well as the Br and Br atoms from the upper and lower atomic planes (*d*_Br:up-Br:down_), after the introduction of (**a**) intrinsic and (**b**) extrinsic defects, compared with the pristine atomic structures. Positive/negative values indicate the expansion/shrinking of the inter-atomic distances.

Regarding 2D materials, research has shown that this magnetic phase transition can also be obtained through hole-doping in 2D group-III oxides and chalcogenides (e.g., GaO, GaSe, and InSe)^18–21^, group-IV monoxides (SnO and PbO)^22–24^, 2D SnX_2_ (X = S and Se)^25^, blue phosphorene, and gray arsenene^26^, etc. Ferromagnetism in these monolayers results from the exchange splitting of electronic states at the top of their valence bands, which exhibit sharp Van Hove singularities in the density of states, resulting in a so-called Stoner instability. Even more 2D candidate materials have been identified using high-throughput calculations with moderate Curie temperatures^27^. It has been predicted that metal vacancy defects and extrinsic *p*-type impurity doping could also lead to a stable ferromagnetic state in some of these 2D materials^21,23,28^. However, the availability of such materials is limited^29,30^, and the generation of shallow acceptor states required for effective p-type doping can be challenging. Therefore, continuous exploration of a broader range of 2D materials that demonstrate ferromagnetism induced by efficient p-type doping is of research significance, as illustrated in this work in the case of PbBr_2_ and HgBr_2_ monolayers.

In this work, we have investigated the stability, electronic, and magnetic properties of 2D (monolayer) PbBr_2_ and HgBr_2_ using spin-polarized first-principles calculations, based on density functional theory. Our findings indicate that although these 2D dihalides are intrinsically nonmagnetic, a stable ferromagnetic phase can be induced for a hole density above a few 10^13^ cm^-2^. The possibility of inducing a hole-doped stable ferromagnetic order in 2D (monolayer) PbBr_2_ and HgBr_2_ through intrinsic and extrinsic point defects is also systematically studied.

## Results

### Atomic structures and stability

Global structural searching, based on evolutionary algorithms using USPEX^31^, was conducted to explore potential 2D atomic structures for the Pb–Br and Hg–Br systems with varying stoichiometries. The results of this search, along with the corresponding convex hull diagrams, are displayed in Fig. 1a and b, respectively. These diagrams plot the enthalpy of formation per atom ($\Delta {H}$) against the composition ratio, allowing for a comparison of the relative stability of different structures and compositions. $\Delta {H}$ can be calculated by

1 $$\begin{aligned} \Delta {H}=\dfrac{E_{M_{x}Br_{y}}-xE_{M}-yE_{Br}}{x+y} \end{aligned}$$

where $E_{M_{x}Br_{y}}$ is the total energy of $M_{x}Br_{y}$ ($M=Pb, Hg$), $E_{M}$ and $E_{Br}$ are the energies of a metal atom and a Br atom in their 2D structures or gas phase found by USPEX. A thermodynamically stable phase is characterized by having a lower formation enthalpy than any other phase of the same composition. The stability of a phase is determined by its position on the convex hull diagram.

**Figure 5:**
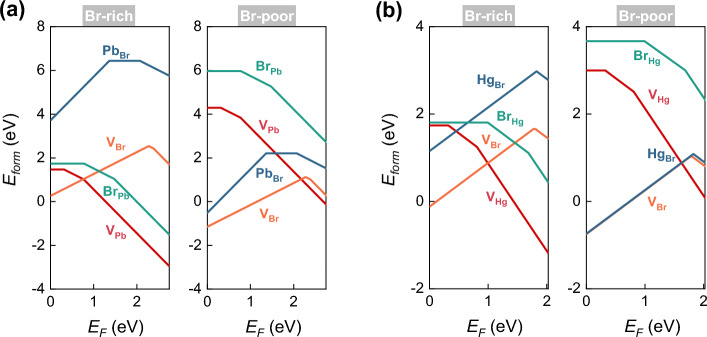
Intrinsic defect formation energies of (**a**) 2D PbBr_2_, and (**b**) 2D HgBr_2_ as a function of the Fermi level within the band gap in the Br-rich and Br-poor limit.

**Figure 6:**
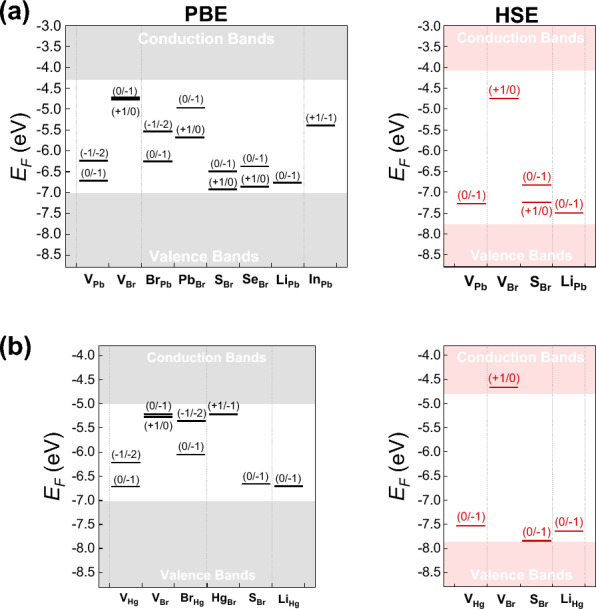
Thermodynamic transition levels for the considered intrinsic and extrinsic defects in (**a**) 2D PbBr_2_, and (**b**) 2D HgBr_2_. The charge transition levels obtained by PBE and HSE06 functionals are given in black and red, respectively. The band edge positions are aligned with respect to the vacuum level.

According to the results, PbBr_2_ and HgBr_2_ are the most stable structures in the Pb–Br and Hg–Br systems, respectively, with their $\Delta {H}$ values of $-\,\,0.98$ eV/atom and $-\,\,0.45$ eV/atom indicated by the red dots on the convex hull diagrams. Some other structures with their formation enthalpies near the convex hulls are provided in Tables S1 and S2, respectively, for the Pb–Br and Hg–Br systems. The atomic structures of 2D PbBr_2_ and HgBr_2_ are similar to those of the 1T 2D transition metal chalcogenides, and they exhibit trigonal symmetry with a space group of P3m1 and a point group of D3d. The metal atoms in these structures are arranged in closed packed planes that are sandwiched between two layers of Br atoms. Each Br atom is bonded to three neighboring metal atoms, while each metal atom is sixfold coordinated. The in-plane lattice constants of 2D PbBr_2_ and HgBr_2_ are calculated to be 4.49 Å and 4.14 Å, respectively, and the Pb–Br and Hg–Br bond lengths are 3.08 Å and 2.87 Å, respectively.

As can be seen in the phonon spectra, all of the phonon branches are positive without any imaginary frequency, suggesting their dynamic stability. In addition, the cleavage energy of 2D HgBr_2_ was calculated by isolating the monolayer from its layered bulk structure using the DFT-D2 Grimme dispersion corrections^32^ and the optB86-vdW functional^33^. The calculated values are 0.240 J/m^2^ and 0.245 J/m^2^ for the two methods, respectively. These values are lower than the cleavage energy of graphene, which is 0.370 J/m^2^^34^, indicating that the exfoliation of 2D HgBr_2_ could be experimentally feasible.

**Figure 7:**
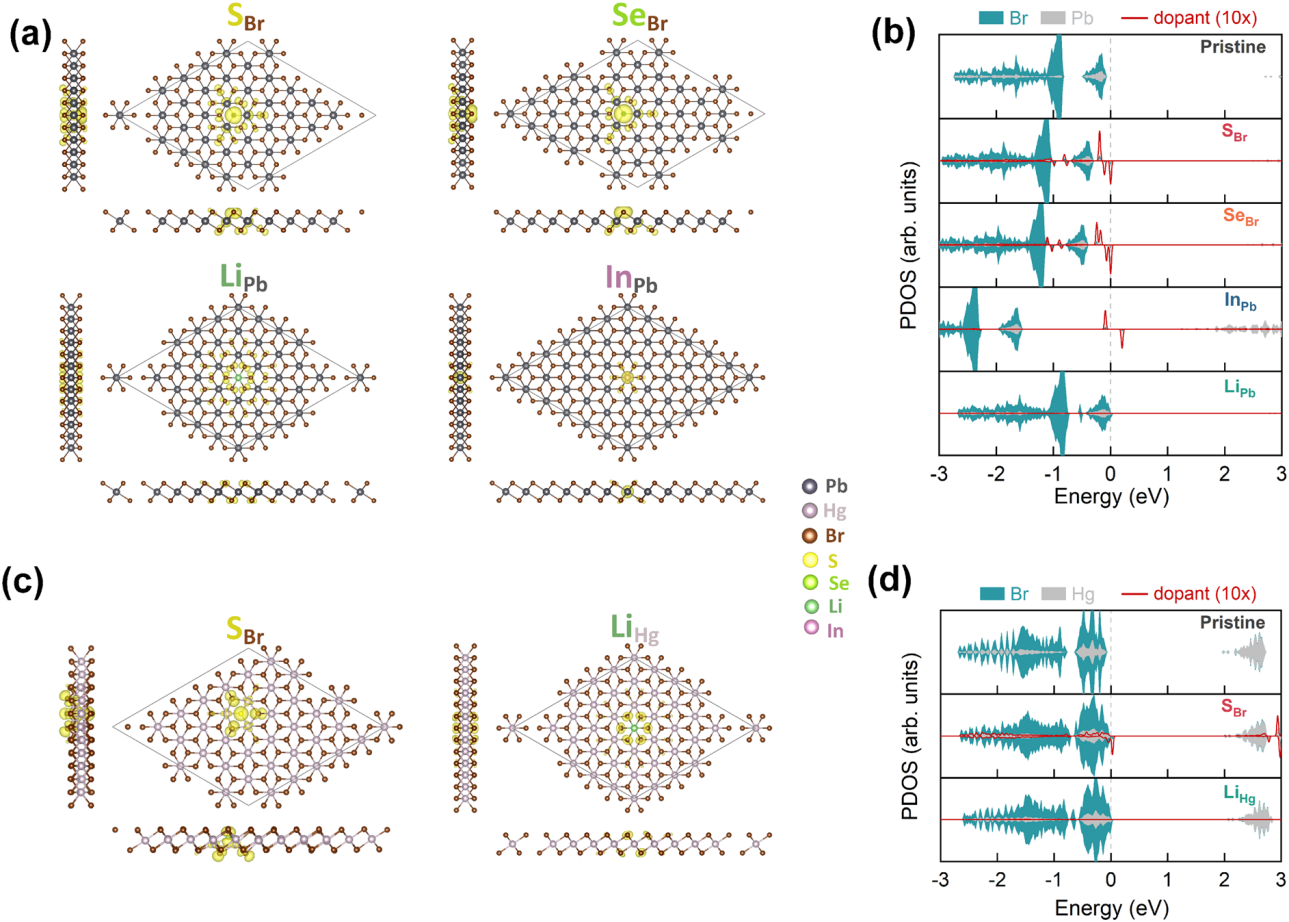
Relaxed atomic structures and Projected density of states of (**a**) and (**b**) 2D PbBr_2_, and (**c**) and (**d**) 2D HgBr_2_, respectively, with different extrinsic defects. The spin densities are shown in yellow (spin-up) and cyan (spin-down). The magnetic moment of these extrinsic defects is 1 $\mu _B$.

**Figure 8:**
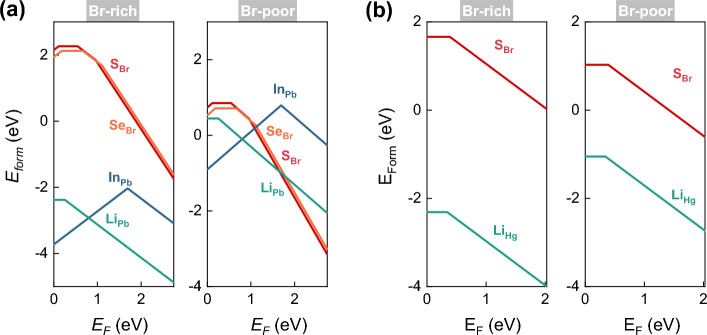
Extrinsic defect formation energies of (**a**) 2D PbBr_2_, and (**b**) 2D HgBr_2_ as a function of the Fermi level within the band gap in the Br-rich and Br-poor limit.

The electronic band structures presented in Fig. 1 demonstrate that both 2D dibromides exhibit intrinsic semiconductor behavior and they are both nonmagnetic. Specifically, 2D PbBr_2_ has an indirect band gap of 2.75 eV at the PBE level, with the top valence band primarily composed of the *p* orbitals of the Br atoms and partially contributed by the 6*s* orbital of the Pb atoms, while the bottom conduction band mainly arises from the Pb-6*p* orbitals. On the other hand, 2D HgBr_2_ exhibits a direct band gap of 2.03 eV at the PBE level, with the top valence band mainly composed of the Br-4*p* orbitals and the bottom conduction band arising from the hybridization of the Hg-6*s* and Br-4*p* orbitals. By employing the HSE06 functional, the band gaps of PbBr_2_ and HgBr_2_ are further increased to 3.69 and 3.07 eV, respectively.

The difference in orbital contributions in the valence band and the conduction band regions, between PbBr_2_ and HgBr_2_, is influenced by the electron configurations of the Pb ([Xe] 4*f*^14^ 5*d*^10^ 6*s*^2^ 6*p*^2^) and Hg ([Xe] 4*f*^14^ 5*d*^10^ 6*s*^2^) atoms and their interactions with the Br atoms. In PbBr_2_, the overlapping of the outmost Pb-6*p* orbitals and Br-4*p* orbitals leads to stable chemical bonds, whereas in HgBr_2_, the involvement of the Hg-6*s* orbitals adds complexity to the bonding mechanism, resulting in the formation of bonding and antibonding states.

### Ferromagnetism induced by hole-doping

The density of states (DOS) of the two 2D dibromides exhibits sharp peaks near the Fermi level due to the relatively flat top valence bands. Hole-doping can cause the Fermi level to approach these peaks, leading to electronic instability that may trigger a nonmagnetic to ferromagnetic phase transition.

Figure 2 demonstrates that hole-doping induces a ferromagnetic order in 2D PbBr_2_ and HgBr_2_, as evidenced by positive spin-polarization energies and magnetic moments of approximately 1.0 $\mu _B$ per hole. The spin-polarization energy, defined as the difference in energy between the nonmagnetic and ferromagnetic states, generally increases with hole density. The spin-polarization energies obtained from the HSE06 and PBE functionals exhibit a relatively large difference. Although direct comparisons with other calculations or experimental data are unavailable, the calculation of the magnetic exchange coupling parameters, which can be calculated by the energy difference between ferromagnetic and antiferromagnetic configurations, suggests that the PBE functional generally underestimates this value, while the results obtained from the HSE06 functional are in better agreement with experimental data, albeit with a slight overestimation^35,36^. Therefore, it is likely that the spin-polarization energies calculated using the HSE06 functional are more accurate, as compared to those obtained from the PBE functional. Furthermore, a hole density of a few 10^13^ cm^-2^ is expected to be sufficient to trigger the non-magnetic to ferromagnetic transition for 2D PbBr_2_ and HgBr_2_ for both functionals. Similar hole densities have been achieved in Mn-doped GaAs and InAs^9,10,37^, as well as in other 2D materials^38,39^, using the back-gate technique. Moreover, a few antiferromagnetic states were constructed and it was discovered that the ferromagnetic states consistently exhibit lower energy levels in these two hole-doped monolayers^27^.

The structural variation and band gaps at different doping densities using PBE and HSE06 functionals are summarized in Table S3. Similar trends are obtained using these two exchange and correlation functionals. The band gaps of 2D PbBr_2_ and HgBr_2_ increase with higher hole density, as long as the materials remain non-magnetic. Upon reaching a certain hole density and undergoing the non-magnetic to ferromagnetic transition, they become half-metallic. In this ferromagnetic state, the spin-down states become metallic, while the spin-up states remain insulating. Notably, the magnetic moments primarily originate from the *p* orbitals of the Br atoms, as evidenced by the spin density plots presented in Fig. S1. Besides, the band gaps of the spin-up channels increase with higher hole doping densities, whereas the spin-down channels exhibit the opposite trend. Additionally, both the metal–Br bond lengths and the Br–Br distances decrease as the hole density increases. It is worth mentioning that the values calculated with the HSE06 functional show a relatively larger extent of structural variation, as compared to those obtained with the PBE functional.

Moreover, exploring the impact of intrinsic and extrinsic defects on *p*-type doping and their potential contribution to the development of ferromagnetism in these 2D materials should be interesting.

### Hole-doping induced by intrinsic defects

Intrinsic defects, including vacancies (V_Pb_, V_Hg_, V_Br_) and antisites (Pb_Br_, Hg_Br_, Br_Pb_, Br_Hg_), were created using a supercell size of $6\times 6\times 1$, which corresponds to a defect density of about $2\times 10$^13^ cm^-2^. The relaxed atomic structures of these defects as well as their spin densities are shown in Fig. 3a and c, respectively, for 2D PbBr_2_ and HgBr_2_. No significant structural reconstruction was observed in the lattice due to the presence of intrinsic defects, and the structural distortions mainly exist around the defects. The biggest changes in inter-atomic distances can be seen by comparing the structures with and without defects, as summarized in Fig. 4. In 2D PbBr_2_ with intrinsic defects, the changes in inter-atomic distances are typically within 5$\%$, except for the Pb–Br bond lengths in V_Pb_ and the Pb–Pb distances in V_Br_, which decrease by 5.3$\%$ and 7.6$\%$, respectively. On the other hand, the atomic structure of 2D HgBr_2_ is more sensitive to the defects, with the Hg-Hg distances in V_Br_ and Hg_Br_, as well as the distance between the Br atoms on the two atomic planes in Br_Hg_, increasing by 11.3$\%$, 5.6$\%$, and 5.6$\%$, respectively.

It is noteworthy that all the intrinsic defects, except for the Br vacancy of 2D HgBr_2_, can lead to net magnetic moments in the two monolayers, which is evident from the spin densities as well as the asymmetric spin-up and spin-down channels in the projected density of states (PDOS) shown on the right-hand side panel of Fig. 3b and d. Specifically, the magnetic moments are 2, 2, 3, 3, 3, 1 and 1 $\mu _B$ for V_Pb_, V_Hg_, Pb_Br_, Hg_Br_, Br_Pb_, Br_Hg_, and V_Br_ (of 2D PbBr_2_), respectively. By comparing the PDOS of the pristine structure to that of the defective structures, it can be observed that the Fermi level shifts towards the conduction band region for Br vacancies and metal antisites, whereas it stays near the top valence band for metal vacancies and Br antisites. This suggests that Br vacancies and metal antisites could act as charge donors, while metal vacancies and Br antisites may act as charge acceptors. Moreover, the defect states are situated near the valence band maximum (VBM) or the conduction band minimum (CBM) for metal vacancies, Hg_Br_, and V_Br_ (in 2D HgBr_2_), while the ones for the remaining defects are relatively deep inside the band gap.

To investigate whether these defects act as shallow or deep acceptors/donors, their formation energies under different charge states are calculated, and the most stable ones across the band gaps are shown in Fig. 5. These formation energies can vary from Br-rich (equivalent to Pb, Hg-poor) to Br-poor (equivalent to Pb, Hg-rich) conditions. In the Br-rich limit, the chemical potential of a Br atom in Br_2_, denoted as $\mu _{Br}$, is used, and the chemical potential of the metal atom (for example, $\mu _{Pb}$) is obtained as $\mu _{Pb}=\mu _{PbBr_{2}}-2\mu _{Br}$. In the Br-poor limit, the chemical potential of a Pb or Hg atom in the bulk metal is used as the reference for $\mu _{Pb}$ or $\mu _{Hg}$, and $\mu _{Br}$ is then obtained by the difference between the chemical potentials of the dibromide and the metal. For example, $\mu _{Br}$ of PbBr_2_ can be obtained by $\mu _{Br}=(\mu _{PbBr_{2}}-\mu _{Pb})/2$ at the Br-poor limit.

Considering the band gap underestimation of the PBE functional, some charge transition levels are also calculated using the HSE06 functional. Both sets of results are summarized in Fig. 6. In addition to the neutral charge state, the negative charge states of metal vacancies and Br antisites are more stable within the band gap, while the positive charge states are more stable for Br vacancies and metal antisites. This further suggests that the former defects could induce *p*-type doping, while the latter could result in *n*-type doping of the two 2D dibromides. Specifically, the (0/− 1) transition levels are 0.31 and 0.32 eV (0.48 and 0.33 eV at the HSE06 level) above the VBMs for V_Pb_ and V_Hg_, respectively. The (0/− 1) transition levels of Br_Pb_ and Br_Hg_ are much deeper inside the band gaps, reaching 0.78 and 0.99 eV above the VBMs, suggesting the deep acceptor nature of Br antisites. The transition levels from + 1 to 0 state for V_Br_ of PbBr_2_ and HgBr_2_ are 0.48 eV (0.69 eV at the HSE06 level) and 0.25 eV (inside the conduction band at the HSE06 level) below the CBMs, respectively. For Pb_Br_ and Hg_Br_, the transition levels from + 1 to 0 or − 1 state are 0.68 eV and 0.2 eV below the CBMs, respectively. These results suggest that Br vacancies and metal antisite defects can act as deep donors in 2D PbBr_2_, while they have shallow donor levels in 2D HgBr_2_.

Figure 5 indicates that the formation energies of metal vacancies and Br antisites are lower under Br-rich conditions, whereas the formation energies of Br vacancies and metal antisites are higher. Consequently, the expected densities of metal vacancies and Br antisites are higher, while the densities of Br vacancies and metal antisites would be suppressed. This could potentially limit the intrinsic *n*-type doping of 2D PbBr_2_ and HgBr_2_. Therefore, to achieve possible *p*-type doping, it is preferred to grow the two 2D dibromides under Br-rich condition.

### Hole-doping induced by extrinsic defects

Regarding extrinsic defects, we investigated the possiblity to substitute a Pb/Hg atom by a Li or In atom, and a Br atom by an S or Se atom, as these impurity atoms have fewer out-most electrons than the metal or Br atoms. Such substitutional impurities should be effective in inducing hole-doping and a subsequent ferromagnetic order in the 2D dibromides. Figure 7 shows the relaxed atomic structures with the spin densities and the corresponding PDOS. Similar to intrinsic defects, there is no apparent structural distortion upon introducing the impurities, with the exception of the elongation of Hg–Br bonds of S_Br_ in HgBr_2_ by 12.8%. The largest structural variations are usually about 5% as compared to the pristine atomic structures. Furthermore, impurity doping results in a magnetic ground state, as reflected by the spin densities and the PDOS, with a total magnetic moment of 1 $\mu _B$.

For Li_Pb_ and Li_Hg_, the magnetic moments are almost exclusively distributed on the neighboring Br atoms, with merely no contribution from the Li or Pb/Hg atoms. On the contrary, the magnetic moments of S_Br_, Se_Br_, and In_Pb_ are distributed on both the impurity and the host atoms. This is evident from their PDOS in Fig. 7b and d, where no defect states near the VBMs are contributed by the Li atom. In contrast, there are spin-up and spin-down states derived from the S, Se, or In dopants, and these states (except for S_Br_ in HgBr_2_) are not close to the VBMs, especially the ones from In, which are in the middle of the band gap.

From the formation energies of PbBr_2_ with extrinsic defects, shown in Fig. 8a, it can be observed that both the + 1 and − 1 charge states of In_Pb_ are stable, with the (+ 1/− 1) transition level lying at about 1.7 eV above the VBM, indicating that this defect is an amphoteric defect rather than a shallow acceptor. As for S_Br_ and Se_Br_, the + 1, 0, − 1, and − 2 charge states are stable in the band gap. The (+ 1/0) levels are 0.11 and 0.18 eV, and the (0/− 1) levels are 0.54 and 0.56 eV above the VBM, respectively, for S_Br_ and Se_Br_. When using the HSE06 functional, the (0/− 1) level of S_Br_ further increases to 0.93 eV above the VBM (Fig. 6a). Therefore, neither S nor Se acts as shallow acceptors in 2D PbBr_2_. On the other hand, Li appears to be a suitable candidate to achieve effective *p*-type doping in 2D PbBr_2_, since its (0/− 1) level is only 0.25 eV (0.27 eV using HSE06 functional) above the VBM. Additionally, under Br-rich conditions, the formation energy of the Li_Pb_ defect is negative at the neutral state, implying the possibility of spontaneous formation.

For S_Br_ and Li_Hg_ in 2D HgBr_2_, only the neutral and − 1 charge states are stable, with their (0/− 1) levels lying at 0.39 and 0.34 eV above the VBM, respectively. Surprisingly, the two levels decrease even further to 0.02 and 0.22 eV from the VBM when using the HSE06 functional, confirming that they are shallow acceptors. Furthermore, the formation energies of these two defects are rather low, as shown in Fig. 8b. Li_Hg_ has a negative formation energy even under Br-poor conditions, and the formation energy of S_Br_ is lower than 2 eV under Br-rich conditions.

## Conclusion

In summary, we performed spin-polarized density functional theory calculations to study the stability, electronic and hole-induced magnetic properties of 2D PbBr_2_ and HgBr_2_, with a focus on the impact of hole-doping induced by intrinsic and extrinsic defects. The results revealed that a hole density of a few 10^13^ cm^-2^ can induce stable ferromagnetic order in these 2D dibromides. These 2D materials are hence potentially interesting as channels in 2D-based spintronic devices, their magnetic state being possibly controlled by e.g. a gate bias. The Br antisite defects behave like deep acceptors, while the acceptor levels from the metal vacancy defects were relatively shallower, with low formation energies under Br-rich conditions. In contrast, both the Br vacancy and metal antisite defects act as deep charge donors in 2D PbBr_2_ or shallow charge donors in 2D HgBr_2_.

Among the doping impurities considered, the substitution of metal atoms by Li is found to produce shallow acceptor levels with very low formation energies. By growing the layers under Br-rich conditions, the concentration of Li impurities should increase while the amounts of Br vacancies and metal antisites should decrease. Additionally, S is found to induce a very shallow acceptor level in 2D HgBr_2_ by replacing a Br atom. Growing this 2D material under Br-poor conditions should result in a large amount of Br vacancies, allowing the doped S atoms to fill the vacant sites and become electrically active.

## Methods

All the spin-polarized DFT calculations were performed using the Vienna ab initio simulation package (VASP)^40,41^ with electron-ion interaction described by PAW pseudopotentials. The GGA, parameterized by the PBE approach^42^ was used as the exchange correlation functional. The energy cutoff of 480 eV and k-point meshes of 0.03$\times$2$\pi$ and 0.02$\times$2$\pi$ Å$^{-1}$ were used for structural optimizations and self-consistent calculations, respectively. Total energy convergence criterion of $10^{-6}$ eV/cell and force convergence criterion of 0.01 eV/Å were chosen. For HSE06 calculations^43^, the energy cutoff of 360 eV and the gamma k-point mesh were used. A Vacuum space larger than 15 Å was used to avoid interactions between the adjacent images in the out-of-plane direction. Hole doping was simulated by selectively removing valence electrons from the system, while maintaining charge neutrality by introducing a jellium background with an opposite charge.

The search for possible 2D structures of lead halide and mercury halide was conducted using an ab initio evolutionary algorithm, implemented in USPEX^31^, in conjunction with the VASP code. The exploration involved variable-composition searching, where the total number of atoms in the 2D crystals was constrained to a range of 2–8. A set of 150 symmetry groups was employed to generate random symmetric structures as the initial population. Subsequently, the full structure relaxations were performed, and the most stable and metastable atomic structures were screened and inherited into the next generation based on their formation enthalpy. The number of generations was set to 40. Structures with formation energies located on the convex hulls are considered the most stable, while those with formation energies above the convex hulls are regarded as metastable structures.

To evaluate the stability of different point defects with respect to the constituents, the formation energy, $E_{form}$ of a defect *D* in charge state *q* can be calculated based on the supercell method^44–46^:

2 $$\begin{aligned} E_{form}(D^q) = E_{tot}(D^q) - E_{tot}(pristine) - \sum _{i}n_i\mu _i + {q(E_F + E_v)} \end{aligned}$$

where $E_{tot}(D^q)$ and $E_{tot}(pristine)$ are the total energies of the supercell with point defects in a dimensionless charge state *q* and the pristine host material, respectively. *q*=0 corresponds to a neutral defect; if one electron is added, *q*= − 1 and if one electron is removed, *q* = + 1. In our calculations, five charge states, i.e., + 2, + 1, 0, − 1 and − 2 are considered and geometry relaxations are carried out for the charged supercells. $\mu _i$ represents the chemical potential of the atom species and $n_i$ is the number of atoms *i* added ($n_i$ > 0) to or removed ($n_i$ < 0) from the pristine material to form the defect. It should be noted that $\mu _i$ is dependent on the experimental growth condition, which can be varied from atom *i*-rich to atom *i*-poor condition. Defects in semiconductors can be assumed in different charge states by exchanging electrons with an electron reservoir. The energy needed in this process for an electron is the electron chemical potential, $(E_F + E_v)$, where $E_F$ is the Fermi level and $E_v$ is the VBM of the pristine material. In finite charge states, the defect formation energy is a function of $E_F$. Generally, one may expect a higher probability for a particular defect with a lower formation energy to be present in the material.

The transition level between two defect charge state $q_1$ and $q_2$, $\varepsilon (q_1/q_2)$, is defined by the value of $E_F$ where the formation energies of the two charge states are equal:

3 $$\begin{aligned} \begin{aligned} E_{tot}(D^{q_1})&- E_{tot}(pristine) - \sum _{i}n_i\mu _i + q_1E_F \\&\quad =E_{tot}(D^{q_2}) - E_{tot}(pristine) - \sum _{i}n_i\mu _i + q_2E_F \end{aligned} \end{aligned}$$

thus

4 $$\begin{aligned} \varepsilon (q_1/q_2)=\dfrac{E_{tot}(D^{q_2})-E_{tot}(D^{q_1})}{q_1-q_2} \end{aligned}$$

The charge transition levels (CTLs) can be obtained by directly comparing the total energies of defective supercells in various charge states, which requires corrections schemes for the convergence of the total energy with respect to the cell size and interactions of the homogeneous background charge^45–48^. As a alternative, the Slater-Janak (SJ) transition state theory can be used to determine the defect charge transition levels without the requirement of comparing the total energies of the systems in different charge states. This method has been applied to successfully evaluate the charge transition levels for GaN^49^, ZnO^50^, LiNbO_3_^51^ and 2D TMDs^52^. In this study, we applied this method to calculate the charge transition levels (CTLs) of the defects in 2D PbBr_2_ and 2D HgBr_2_.

The SJ method states that the *i*th Kohn-Sham eigenvalue $\varepsilon _{i}$ is related to the derivative of the total energy *E* with respect to the occupancy number $\eta _i$ of the respective orbital^53^,

5 $$\begin{aligned} \dfrac{\partial E}{\partial \eta _i}=\varepsilon _i \end{aligned}$$

Assuming a linear dependency of the eigenvalue for the highest occupied state $\varepsilon _H$ on the occupation number^54^, the electron affinity level can be obtained as

6 $$\begin{aligned} E^{N+1}-E^N=\int _{0}^{1}\varepsilon _H d\eta =\varepsilon _H \Big (\frac{1}{2} \Big ) \end{aligned}$$

Therefore, the transition level between $q_1$ and $q_2=q_1\pm 1$ can be written as

7 $$\begin{aligned} \varepsilon (q_1/q_2)= {\left\{ \begin{array}{ll} \varepsilon _H \left( q_1+\frac{1}{2};R_{q_1}\right) -\lambda _{q_2}, &{} q_2=q_1 +1 \\ \varepsilon _H \left( q_1-\frac{1}{2};R_{q_1}\right) +\lambda _{q_2}, &{} q_2=q_1 -1 \\ \end{array}\right. } \end{aligned}$$

Where $R_{q_1}$ represents the configuration in charge state $q_1$, $\lambda$ is the reorganisation energy which is obtained by the total energy difference between equal charge state but in different configurations:

8 $$\begin{aligned} \lambda _{q_2}=E_{tot}\left( q_2;R_{q_2}\right) -E_{tot}\left( q_2;R_{q_1}\right) \end{aligned}$$

Because of the ionic relaxations of the charge defect supercells, the calculated transition levels should be corrected with the reorganization energies to reduce the energy cost for the transition with respect to the Fermi level^29^.

In our calculations, $\varepsilon _H$ of the (partially) charged defect supercells as well as the pristine 2D layer are referenced relative to the electrostatic potential averaged over the PAW sphere of a specific atom which is far away from the defect sites (typically larger than 15 Å). In this way, the Kohn-Sham eigenvalue of the defective supercell can be referenced to the VBM of the pristine host material. Benchmark calculations were performed on the native defects of a MoS_2_ monolayer, using the SJ method. The calculations yielded a (0/− 1) level of approximately 0.8 eV for the Mo vacancy and a (0/+ 1) level of about 1.45 eV for the S vacancy. These results are in excellent agreement with previously computed results which are based on the total energy method^55^.

## Supplementary Information

Supplementary Information 1.

## Data Availability

The datasets used and/or analysed during the current study are available from the corresponding author on reasonable request.
