# Supplementary material for: Ferromagnetism in two-dimensional metal dibromides induced by hole-doping
Source: Sci Rep. 2023 Jul 17;13:11521. doi: 10.1038/s41598-023-37777-8 (PMC10352248; doi:10.1038/s41598-023-37777-8)
Supplement: Supplementary file 1 — Supplementary Information 1. [file 41598_2023_37777_MOESM1_ESM.pdf]

Supplementary Information for

Ferromagnetism in two-dimensional metal dibromides induced by hole-doping

Ruishen Meng[1,*] **and Michel Houssa**[1,2,*]
[1]KU Leuven, Department of Physics and Astronomy, Leuven, B-3001, Belgium
[2]imec, Leuven, B-3001, Belgium
[*]ruishen.meng@kuleuven.be
[*]michel.houssa@kuleuven.be

Table S1. Atomic structures and properties of 2D PbBr2 candidates found in the USPEX searching process on or slightly above the convex hull. The total energy per atom, energy difference compared to the most stable structure, top view and side view representations, atomic coordinates (in POSCAR format), and the results of hole doping simulations at the PBE level for selected candidates, are provided.

**2D $PbBr_2$**				
**Total energy:**	-3.230 eV/atom	-3.198 eV/atom	-3.166 eV/atom	-3.151 eV/atom
**Energy difference:**	0 eV/atom	0.032 eV/atom	0.064eV/atom	0.080 eV/atom
**Atomic structure (Top view & side view)**	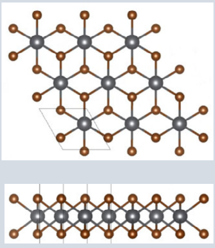	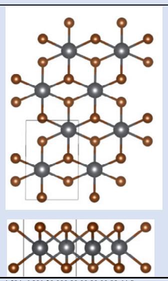	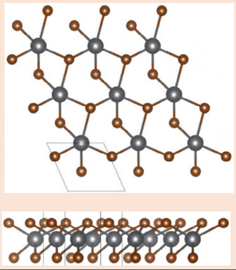	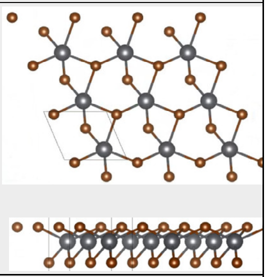
**POSCAR**	EA739 4.469 4.465 20.000 90.00 90.00 119.94 Sym.group: 164<br>1.0<br>4.402746 -0.768765 0.000000<br>-1.529989 4.194864 0.000000<br>0.000000 0.000000 20.000000<br>Pb Br<br>1 2<br>Direct<br>0.766637 0.624920 0.500246<br>0.099358 0.288562 0.583229<br>0.432171 0.955594 0.416525	EA941 4.584 6.901 20.000 90.00 90.00 89.64 Sym.group: 12<br>1.0<br>4.581087 -0.170850 0.000000<br>0.300222 6.894380 0.000000<br>0.000000 0.000000 20.000000<br>Pb Br<br>2 4<br>Direct<br>0.808844 0.881601 0.501262<br>0.308376 0.380898 0.498686<br>0.309751 0.029580 0.588751<br>0.808224 0.233075 0.411341<br>0.311781 0.738393 0.412039<br>0.806368 0.524067 0.587920	EA2571 4.904 4.563 20.000 90.00 90.00 113.78 Sym.group: 1<br>1.0<br>4.903715 -0.008128 0.000000<br>-1.833200 4.178458 0.000000<br>0.000000 0.000000 20.000000<br>Pb Br<br>1 2<br>Direct<br>0.605660 0.994626 0.503580<br>0.036795 0.713732 0.573273<br>0.388999 0.408915 0.423146	EA2043 5.173 4.405 20.000 90.00 90.00 112.95 Sym.group: 1<br>1.0<br>5.172216 -0.071827 0.000000<br>-1.661146 4.079918 0.000000<br>0.000000 0.000000 20.000000<br>Pb Br<br>1 2<br>Direct<br>0.704075 0.221623 0.509670<br>0.143888 0.945370 0.569637<br>0.553750 0.657318 0.420693
**Hole doping simulation (PBE)**			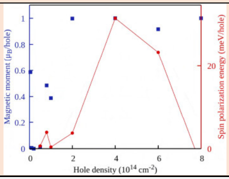	
**Total energy:**	-3.135 eV/atom	-3.119 eV/atom	-3.102 eV/atom	-3.086 eV/atom
**Energy difference:**	0.095 eV/atom	0.111 eV/atom	0.128 eV/atom	0.144 eV/atom
**Atomic structure (Top view & side view)**	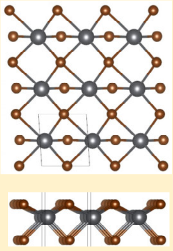	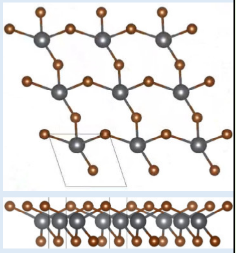	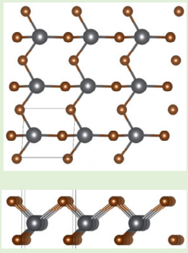	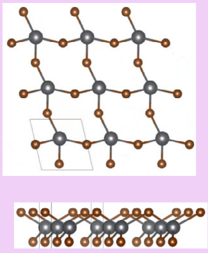
**POSCAR**	EA404 4.122 4.459 20.000 90.00 90.00 93.58 Sym.group: 1<br>1.0<br>4.120286 -0.120378 0.000000<br>-0.148695 4.456445 0.000000<br>0.000000 0.000000 20.000000<br>Pb Br<br>1 2<br>Direct<br>0.095531 0.490700 0.514246<br>0.531140 0.003741 0.573672<br>0.574146 0.505560 0.412083	EA2651 5.117 4.677 20.000 90.00 90.00 108.32 Sym.group: 1<br>1.0<br>5.117086 0.019369 0.000000<br>-1.486653 4.434846 0.000000<br>0.000000 0.000000 20.000000<br>Pb Br<br>1 2<br>Direct<br>0.408267 0.788708 0.506585<br>0.535567 0.309110 0.422509<br>0.935308 0.996789 0.570906	EA1071 4.511 4.273 20.000 90.00 90.00 86.46 Sym.group: 1<br>1.0<br>4.510589 0.002140 0.000000<br>0.261593 4.264874 0.000000<br>0.000000 0.000000 20.000000<br>Pb Br<br>1 2<br>Direct<br>0.217236 0.453375 0.492703<br>0.774765 0.439913 0.587793<br>0.909890 0.971744 0.419505	EA1777 5.049 5.090 20.000 90.00 90.00 103.31 Sym.group: 5<br>1.0<br>5.042853 0.246030 0.000000<br>-1.412235 4.890424 0.000000<br>0.000000 0.000000 20.000000<br>Pb Br<br>1 2<br>Direct<br>0.493993 0.621089 0.499200<br>0.368279 0.119860 0.428679<br>0.995252 0.505223 0.572121
**Hole doping simulation (PBE)**	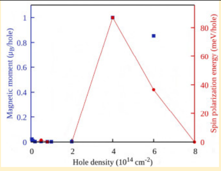	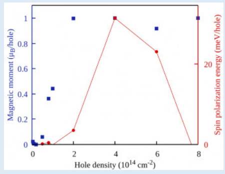		

Table S2. Atomic structures and properties of 2D Hg_2Br_2 and Hg_2Br_2 candidates found in the USPEX searching process on or slightly above the convex hull. The total energy per atom, energy difference compared to the most stable structure, top view and side view representations, atomic coordinates (in POSCAR format), and the results of hole doping simulations at the PBE level for selected candidates, are provided.

<table>
<tr><th colspan="4">2D HgBr2 and Hg2Br2</th></tr>
<tr><td>Total energy:</td><td>-1.573 eV/atom</td><td>1.542 eV/atom</td><td>-1.523 eV/atom</td></tr>
<tr><td>Energy difference:</td><td>0 eV/atom</td><td>0.031 eV/atom</td><td>0.050 eV/atom</td></tr>
<tr><td>Atomic structure (Top view & side view)</td><td>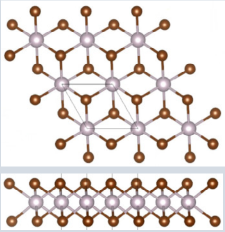</td><td>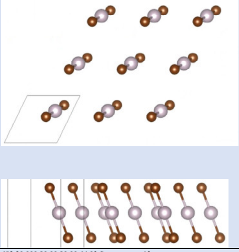</td><td>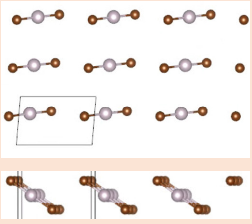</td></tr>
<tr><td>POSCAR</td><td>EA3267 4.141 4.141 20.000 90.00 90.00 119.94 Sym.group: 164
1.0
4.141412 0.000000 0.000000
-2.070701 3.586566 0.000000
0.000000 0.000000 20.000000
Hg Br
1 2
Direct
0.000000 0.000000 0.5739010
0.333333 0.666667 0.6532881
0.666667 0.333333 0.4945144</td><td>EA754 4.682 4.682 20.000 90.00 90.00 64.03 Sym.group: 12
1.0
4.682209 -0.042339 0.000000
2.088264 4.190947 0.000000
0.000000 0.000000 20.000000
Hg Br
1 2
Direct
0.672522 0.672522 0.500236
0.550321 0.550321 0.611249
0.791307 0.791307 0.388514</td><td>EA1334 6.997 4.459 20.000 90.00 90.00 85.24 Sym.group: 2
1.0
6.996622 -0.006667 0.000000
0.374326 4.443525 0.000000
0.000000 0.000000 20.000000
Hg Br
1 2
Direct
0.144675 0.648040 0.499519
0.425187 0.695543 0.431538
0.868722 0.589613 0.568943</td></tr>
<tr><td>Hole doping simulation (PBE)</td><td></td><td></td><td></td></tr>
<tr><td colspan="4"></td></tr>
<tr><td>Total energy:</td><td>-1.295 eV/atom</td><td>-1.279 eV/atom</td><td>-1.263 eV/atom</td></tr>
<tr><td>Energy difference:</td><td>0.279 eV/atom</td><td>0.294 eV/atom</td><td>0.310 eV/atom</td></tr>
<tr><td>Atomic structure</td><td>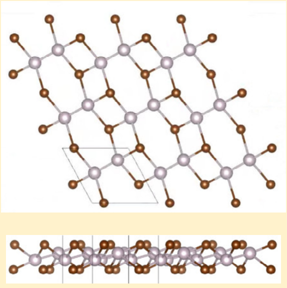</td><td>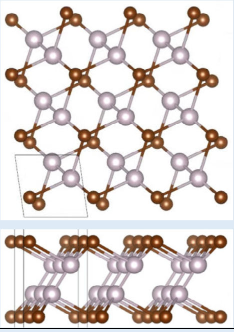</td><td>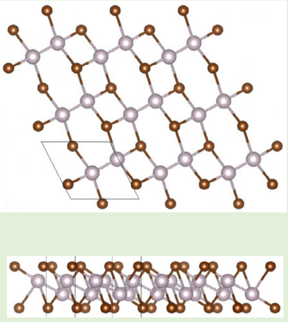</td></tr>
<tr><td>POSCAR</td><td>EA2541 6.289 6.012 20.000 90.00 90.00 118.24 Sym.group: 12
1.0
6.266957 -0.522327 0.000000
-2.394996 5.514234 0.000000
0.000000 0.000000 20.000000
Hg Br
2 2
Direct
0.286684 0.556033 0.485849
0.744149 0.791332 0.517392
0.178597 0.004963 0.558069
0.863006 0.347582 0.438690</td><td>EA1426 4.312 4.221 20.000 90.00 90.00 98.31 Sym.group: 12
1.0
4.311676 -0.027404 0.000000
-0.583418 4.180275 0.000000
0.000000 0.000000 20.000000
Hg Br
2 2
Direct
0.567721 0.886594 0.449568
0.825957 0.624583 0.549940
0.262553 0.207060 0.372465
0.136132 0.316580 0.628028</td><td>EA2467 5.617 5.372 20.000 90.00 90.00 117.14 Sym.group: 2
1.0
5.617207 0.001041 0.000000
-2.451900 4.780251 0.000000
0.000000 0.000000 20.000000
Hg Br
2 2
Direct
0.511957 0.459967 0.527414
0.981621 0.695781 0.471649
0.072028 0.263107 0.589241
0.426652 0.920372 0.411695</td></tr>
<tr><td>Hole doping simulation (PBE)</td><td>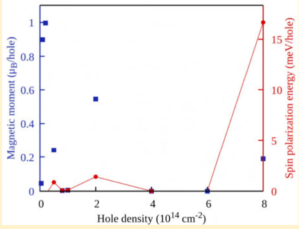
</td><td>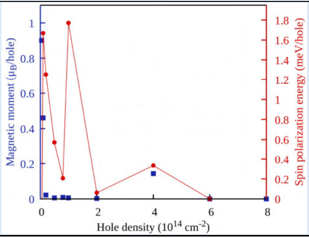
</td><td></td></tr>
</table>

Table S3. Energy band gaps, metal-Br bond lengths, and Br-Br distances at various doping densities for 2D $PbBr_2$. The calculated enegy band gaps of spin-up and spin-down channels ($E_{gap\ (spin\text{-}up)}$, $E_{gap\ (spin\text{-}down)}$), the metal-Br bond length ($d_{(M\text{-}Br)}$) and the distances between Br and Br atoms from the upper and lower atomic planes ($d_{(Br\text{-}Br)}$), using HSE06 and PBE functionals, are provided. It should be noted that the materials exhibit half-metallicity, with the spin-down states becoming metallic while the spin-up states remain insulating when they become ferromagnetic.

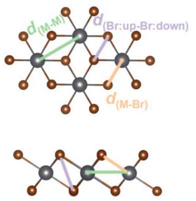

2D PbBr2

	HSE06				PBE			
ρ (×10^{14} cm^{-2})	$E_{gap\ (spin\text{-}up)}$ (eV)	$E_{gap\ (spin\text{-}down)}$ (eV)	$d_{(Pb\text{-}Br)}$ (Å)	$d_{(Br\text{-}Br)}$ (Å)	$E_{gap\ (spin\text{-}up)}$ (eV)	$E_{gap\ (spin\text{-}down)}$ (eV)	$d_{(Pb\text{-}Br)}$ (Å)	$d_{(Br\text{-}Br)}$ (Å)
0.05	3.666	3.666	3.080	4.222	2.742	2.742	3.080	4.222
0.1	3.669	3.669	3.057	4.156	2.773	2.773	3.078	4.218
0.2	3.672	3.672	3.054	4.148	2.751	2.751	3.076	4.212
0.5	3.669	3.534	3.045	4.120	2.764	2.764	3.071	4.195
0.8	3.682	3.474	3.042	4.110	2.782	2.753	3.066	4.181
1	3.698	3.444	3.038	4.099	2.770	2.741	3.064	4.175
2	3.764	3.323	3.024	4.058	2.847	2.729	3.056	4.152

2D HgBr2

	HSE06				PBE			
ρ (×10^{14} cm^{-2})	$E_{gap\ (spin\text{-}up)}$ (eV)	$E_{gap\ (spin\text{-}down)}$ (eV)	$d_{(Hg\text{-}Br)}$ (Å)	$d_{(Br\text{-}Br)}$ (Å)	$E_{gap\ (spin\text{-}up)}$ (eV)	$E_{gap\ (spin\text{-}down)}$ (eV)	$d_{(Hg\text{-}Br)}$ (Å)	$d_{(Br\text{-}Br)}$ (Å)
0.05	3.086	3.086	2.844	3.900	2.027	2.027	2.870	3.975
0.1	3.080	3.060	2.844	3.898	2.056	2.056	2.870	3.975
0.2	3.104	3.025	2.843	3.897	2.061	2.061	2.870	3.975
0.5	3.147	2.996	2.841	3.890	2.074	2.074	2.869	3.970
0.8	3.196	2.974	2.840	3.887	2.113	2.113	2.869	3.970
1	3.203	2.944	2.839	3.886	2.146	2.146	2.868	3.968
2	3.326	2.878	2.839	3.883	2.210	2.137	2.868	3.968

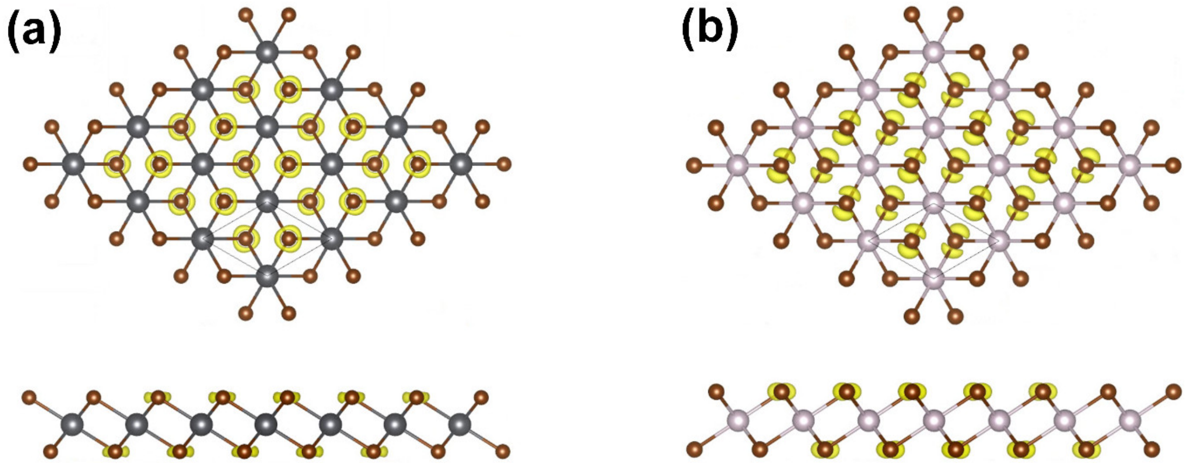

Figure S1. Spin density plots calculated by HSE06 functional for (a) $PbBr_2$ and (b) $HgBr_2$ monolayers at hole density of 2 × 10^{14} cm^{-2}.
